# The Value of Expert Testimony in Medico-Legal Cases from a Medical Standpoint

**Published:** 1898-10

**Authors:** 


### The Value of Expert Testimony in Medico-Legal Cases From a Medical Standpoint.

In the January number of the *Buffalo Medical Journal*, Dr. A. W. Henckell treats the subject as follows:

"The mode of examining experts is, usually, in the form of a hypothetical question, put and paid for by the side calling the experts; in other words, the professional man testifies to certain opinions (which a man can change at will) for the benefit of the highest bidder. Has the medical fraternity reached so low a level as to forget the duties it owes to truth and veracity?

"The expert should be called for the purpose of assisting the law in the obtaining of justice for the prisoner, to assist the court and elucidate certain points to the jury. The hypothetical question has many objections urged against it. Hornblower says:

"'It is claimed, and with much truth, that the hypothetical question assumes as proved whatever the counsel putting the question has endeavored to prove and combines insignificant with important circumstances and alleged facts, supported by slight and perhaps worthless testimony, with other facts of which the proof is strong and convincing, while omitting still other facts of equal or greater importance which may be overwhelmingly established by the other side.'

"Expert testimony based upon such one-sided hypothetical questions is almost of necessity favorable to the questioner, and the true remedy is that already adopted by the laws of our State in insanity cases—the appointment of a commission of experts.

"The primary object of expert testimony is not to prove opinions, but facts, in the shape of rules of science or art, generally recognized by those who are especially instructed in such art or science.

"Lord Campbell stated in the Tracy Peerage case, that 'skilled witnesses came with such a bias on their minds, to support the cause in which they embark, that hardly any weight should be given to their evidence.'

"Judge Davis, of the Supreme Court of Maine, stated: 'If there is any kind of testimony that is not only of no value but often worse than that, it is, in my judgment, that of medical experts.'

"From this it will appear how necessary it is for medical experts to free themselves from all bias and swear only to the truth, the whole truth and nothing but the truth, without fear or favor.

"These opinions are not always merited, and, if medical opinions were free from bias, such opinions would be of inestimable value, as they are in Germany and France, where the expert becomes an official and a component part of the judicial system. The objection to the expert in this country is that parties pay and retain their own experts. The question of the compensation of the medical expert is one which has attracted the attention of the profession. Although most writers on medical jurisprudence make mention of the fact that they should, or must, receive remuneration, the question, I believe, has never been decided in this State. I have the opinion of one of the justices of the supreme court to the effect that the question has never been judicially settled. He stated, as a reason, that no physician would risk being fined for contempt, but would rather answer the question.

"In the recent Carlyle Harris trial, the most eminent experts in the State (and out of it) were called to give evidence. These men testified to facts, so-called, but they were diametrically opposed to each other. Of what value was this testimony in the obtaining of justice?

"Again, in a recent murder trial in New York, experts were on the stand day after day, for the purpose of deciding the question whether or not the presence of human blood could be positively determined; but here again it was of no value to either side.

"In the Benham case the medical expert testimony was again brought into ridicule, to such an extent that the jury practically ignored the testimony of all the experts. The reason for this was very apparent, in that the jurors were not familiar with chemistry and its intricate technicalities. It is even asserted that while an eminent expert was giving his testimony a number of jurors fell into temporary somnolency.

"Again, in the Luetgert case, we found the experts at war with each other and exposed to the ridicule of the public. The aptness of some expert witnesses in murder trials was exemplified in that trial. One of the leading osteologists for the defense, on being shown the skull of a mammal, pronounced it as being the skull of a dog, describing in minutest detail its complex structure. After finishing his explanation, the prosecuting attorney informed him that it was the skull of a monkey. Recognizing the pit he had fallen into, he humorously remarked that it was probably a monkey-faced dog.

"Considering the various phases of expert testimony, one can readily see that a remedy must be sought for and speedily put into practice, if the honored status of the medical profession is to be maintained. In my opinion there are two ways out of the difficulty—the first, the appointment of a commission by the court (I have already alluded to it in the early part of my paper); the other, the appointment of a commission of experts to receive the contentions of the experts of each side, and they to decide and present their finding of facts to the jury, and not to be subject to review. In my opinion, the latter plan is freer from criticism."—*New York Medical Journal.*

